# Evaluation of the effects of different photosensitizers used in antimicrobial photodynamic therapy on tooth discoloration: spectrophotometric analysis

**DOI:** 10.1007/s10103-024-04085-0

**Published:** 2024-05-21

**Authors:** Özge Hür Şahin, Hulde Korucu, Zeliha Uğur Aydin

**Affiliations:** Department of Endodontics, Gulhane Faculty of Dentistry, University of Health Sciences, Ankara, Turkey

**Keywords:** Antimicrobial photodynamic therapy, Endodontics, Phthalocyanine, Tooth discoloration, Zinc phthalocyanine

## Abstract

**Background:**

Tooth discoloration is a common concern in antimicrobial photodynamic therapy (aPDT) using various photosensitizers (PS). Toluidine Blue (TB), Methylene Blue (MB), Phthalocyanine (Pc), and 2-mercaptopyridine-substituted zinc phthalocyanine (TM-ZnPc) are among those studied, but their relative impacts on tooth discoloration remain unclear.

**Aim:**

This study aimed to compare the effects of TB, MB, Pc, and TM-ZnPc in aPDT on tooth discoloration, utilizing a controlled experimental setup.

**Materials and Methods:**

The study comprised seventy-five single-rooted incisors with root canals. Following meticulous preparation, a standardized area on the crown surface was designated for examination, and precise measurements of the initial tooth colors were recorded. Samples were randomly divided into five groups: Negative control, MB, TM, Pc, and TM-ZnPc. Photoactivation was performed using LED light, and color measurements were taken at multiple time points up to 90 days. Data were converted to Lab* color values of the CIE Lab* color system (International Commission on Illumination, Vienna, Austria), and ΔE values were calculated. Statistical analysis was performed using Two-way ANOVA and Post-Hoc Tukey tests (p < 0.05).

**Results:**

At day 7 and 30, TM-ZnPc and Pc caused less discoloration compared to MB and TB. TM-ZnPc caused more tooth discoloration compared to Pc (p < 0.05). Compared to baseline, MB and TM-ZnPc caused more tooth discoloration at 30 days and TB caused more tooth discoloration at 90 days (p < 0.05). No significant difference was observed in terms of tooth discoloration at all periods evaluated after Pc application (p > 0.05). All photosensitizers tested in the study caused tooth coloration.

**Conclusion:**

All PS induced clinically detectable tooth discoloration, with TB and MB causing more significant discoloration compared to Pc and TM-ZnPc at certain time points. TM-ZnPc and Pc demonstrated more stable coloration levels over time, suggesting their potential reliability in aPDT applications. This study highlights the importance of selecting appropriate PS to minimize tooth discoloration in aPDT, with Pc showing promise in this regard.

## Introduction

The application of different photosensitizers (PSs) in antimicrobial photodynamic therapy (aPDT) poses a disadvantage, particularly in the anterior teeth, due to their potential to induce tooth discoloration. The goal of endodontic therapy is to eradicate the pathogenic microflora within the root canal systems, which significantly contributes to the development of pulpitis and apical periodontitis [1]. Due to complex anatomical structures such as the lateral canal, isthmus, accessory canal, and dentinal tubules, disinfection of root canals cannot always be performed at an optimal level with traditional treatment procedures [2] To improve intracanal disinfection, additional disinfection procedures are therefore needed. In addition to traditional methods to improve the disinfection of the root canal system, aPDT application is one of the alternative treatment methods. In aPDT, PSs are activated by light of appropriate wavelength, and antimicrobial activity is provided by hydroxyl (OH-), superoxide (O2-) or lipid-derived radicals formed as a result of the reaction [2, 3].

There are many PSs with different contents, methylene blue (MB) and toluidine blue (TB) in the phenothiazine group are the most common PSs used for additional disinfection in endodontics due to their long history of use, cheapness, easy availability, stable compounds, and low toxicity [4, 5]. In the literature, it has been reported that the intracanal microbial load decreased significantly after aPDT using MB and TB [6]. The low-intensity absorption of the PSs in the phenothiazine group, however, increases their penetration into the dentinal tubules, causing them to be difficult to remove after application and leading to tooth discoloration, which poses aesthetic problems [7]. Due to increasing aesthetic concerns growing today, the search for alternative PS continues to overcome the problem of tooth discoloration [8]. In vitro studies have demonstrated the efficacy of phthalocyanine (Pc) as a promising root canal irrigant against endodontic microorganisms; however, there is currently no literature available on the tooth discoloration potential associated with Pc. To increase the effectiveness of Pc, different types of PS are produced by adding different metal ions to its content. Zinc phthalocyanine (TM-ZnPc) is particularly promising for aPDT due to its favorable characteristics, including excellent light stability, strong absorption within the phototherapeutic window (600–900 nm), efficient production of singlet oxygen, high phototoxicity, and minimal toxicity in the absence of light exposure [9–11]. Compared to other PSs, tetrasubstituted phthalocyanine derivatives have high water solubility, enabling them to be metabolized quickly in the organism and increase their bioavailability [12–14].

The literature review revealed promising outcomes for Pc and TM-ZnPc in decreasing intracanal microbial load compared to alternative root canal irrigants [15, 16]. The color change potential of these alternatives relative to MB and TB, however, remains largely unexplored in the existing literature. Therefore, this study aims to determine the potential effect of TB, MB, Pc, and TM-ZnPc on tooth discoloration. The null hypothesis of the study was that there would be no significant difference between the different PSs tested in terms of tooth discoloration.

## Materials and methods

The study was approved by the University of Health Sciences Scientific Research and Publication Ethics Committee (no: 2022 − 361). The sample size was calculated using G*Power software 3.1.2 (Universitat, Düsseldorf, Germany) with 0.05 alpha error probability and 80% power (effect size = 0.25) regarding a study in the literature with a similar design [17]. Power analysis showed that a minimum of 15 samples per group and a total of 75 samples were statistically necessary. This study examined a total of 75 human teeth with single roots and root canals that were extracted due to periodontal issues. The teeth comprised 21 maxillary central incisors, 18 maxillary lateral incisors, 4 maxillary canines, 13 mandibular central incisors, 14 mandibular lateral incisors, and 5 mandibular canines. Teeth extracted for periodontal reasons from individuals aged 35–52 were used in this study. There was no caries in the crown of any of the teeth included.

The debris and soft tissue residues on the root surface of the teeth were removed with a periodontal curette (Golgran-Millennium, Sao Paulo, SP, Brazil). The teeth were stored in 0.1% thymol solution at 4 ◦C for 6 months after extraction until used in the study [18]. The endodontic access cavity was prepared under water cooling using a high-speed handpiece and diamond bur (G&Z Instrumente, Lustenau, Austria). Following the confirmation of apical patency using a #10 K file (VDW, Munich, Germany), the working length of the tooth was established to be 1 mm shorter than the apical foramen. All teeth were prepared with a Resiproc Blue #25 (VDW, Munich, Germany) rotary file system using the torque and rpm specified in the manufacturer’s instructions. Following each file change, the root canals were irrigated with 2 mL of 2.5% sodium hypochlorite (NaOCl) solution (Imicryl, Konya, Turkey). For the final irrigation procedure, 2 mL of 2.5% NaOCl (Imıcryl), 2 mL 17% EDTA (Imicryl, Konya, Turkey), and 2 mL distilled water were used [6]. In all irrigation procedures, a 30 G NaviTip (Ayset, Adana, Türkiye) side perforated irrigation needle was used and placed in the root canal 1 mm shorter than the working length. Root canals were dried with paper points (VDW, Munich, Germany) [19–24]. In this study, an LED light source (630 nm wavelength, power density of 2–4 mW/cm²) was utilized due to its ease of use and its ability to produce light that matches the activation spectrum of all five PSs tested in the study.

### Division of samples into experimental groups and application of the aPDT procedures

The teeth were numbered from 1 to 75. Randomization was accomplished by using computer sequence generation (www.random.org), which provided by a table for 5 groups with randomized tooth numbers in each group (*n* = 15);

**Negative control (NC):** No PS was applied to the teeth in this group.

**MB:** A total of 1 ml of 0.01% (0.1 mg/ml) MB (Merck KgaA, Darmstadt, Germany) was applied to each root canal.

**TB:** 1 ml of 0.1 mg/ml TB (Merck KgaA, Darmstadt, Germany) was applied in each root canal.

**Pc:** 1 ml of 6 micromolar Pc (1 mL (6µM)) was applied to each root canal.

**TM-ZnPc:** (4)-tetrakis-[(2-mercaptopyridine) phthalocyaninato] zinc was synthesized and purified according to the procedures in the literature [12, 25]. 1 ml of 6 micromolar phthalocyanine (1 mL (6µM)) was applied to each root canal [26].

### Photoactivation

**Fig. 1:**
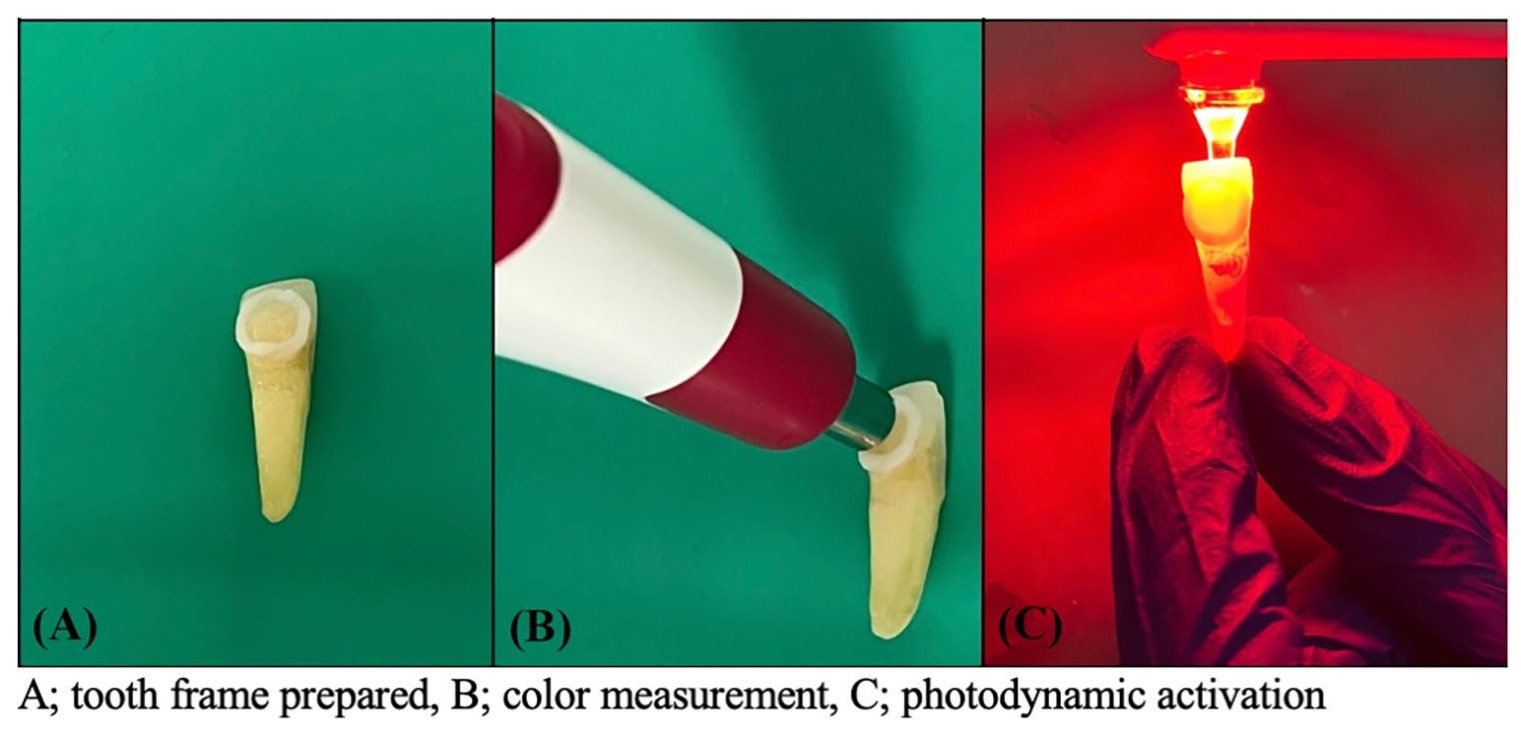
Procedure phases

Except for the negative control in all groups, the pre-irradiation time of PSs was determined as 5 min and LED (Fotosan 630; CMS Dental, Denmark) and Endo tip (diameter 1 mm ^2^) (CMS Dental) were used for photoactivation (Fig. 1, C). The tip of the device was inserted 1 mm shorter than the working length into the root canal and the LED was applied for 60 s with a light wavelength of 620–640 nm (85%), a peak of 630 nm, and an intensity of 2 mW/cm^2^ (Table 1). This protocol was implemented based on previous studies in the literature [6, 27]. The root canals were irrigated with 5 ml of sterile distilled water to remove residual PSs.

**Table 1:** Parameters for aPDT

Concentration of MB	0.1 mg/ml
Concentration of TB	0.1 mg/ml
Concentration of Pc	1 mL (6µM)
Concentration of TM-ZnPc	1 mL (6µM)
Pre-irradiation Time of PS	5[min]
Application Time of LED	1[min]
Output Power of LED	4000 mW/cm^2^
Wavelenght Spectrum of LED	620–640[nm]
Peak of LED	630[nm]

### Tooth color assessment

The color change of the teeth over time was measured with Vita Easyshade Advance (VITA Zahnfabrik, Bad Sackingen, Germany). Vita Easyshade Advance (VITA Zahnfabrik) is the spectrophotometer of choice for reliable color measurement, which has standards of the international CIE L*a*b* color system (International Commission on Illumination) [28]. Before each color assessment procedure, the device was calibrated using the calibration table by the manufacturer’s instructions. To ensure consistent measurements, a fixed frame was prepared using an acrylic model and flowable composite (Tokuyama, Tokyo, Japan), replicating the tip of the spectrophotometer on the buccal surface of the teeth at the 3 mm coronal to the cementoenamel junction (CEJ) (Fig. 1, A). This method aimed to enhance the reliability of color evaluation [29]. During the assessments, the head of the spectrophotometer was placed in the composite frame and the measurement was made (Fig. 1, B). Color measurements were performed three times from the same spot under white light by placing the tip of the spectrophotometer in the frame on the labial surfaces of the teeth. L*, a*, and b* values were obtained for each measurement and the average of these measurements was calculated. L* values indicate color change ranging from black (0) to white (100), a * values from red (+ 80a *), green (− 80a *), and b * values from yellow (+ 80b *) to blue (− 80b *) ) denotes varying color variations [30].

Color measurements were performed with Vita Easyshade Advance (VITA Zahnfabrik, Bad Sackingen, Germany) at 5 different times, as indicated below:

**T0:**

before the processing.

**T1:**

immediately after the dyes used in PS are removed.

**T7:**

1 week after the procedure.

**T30:**

1 month after the procedure.

**T90:**

3 months after the procedure.

The mean color change (ΔE) value was calculated using the following formula [31].:

ΔE*= ((ΔL*) ²+(Δa*) ²+(Δb*) ²) ½

ΔL*=L1*-L0*; Δa*=a1*-a0*; Δb*=b1*-b0*

If the ΔE value was 3.3 ≥, it was accepted that there was a visible color change [31].

Tooth discoloration assessment and aPDT procedure were conducted by the same researcher.

### Statistical analysis

Shapiro-Wilk test was applied to confirm the normality of the obtained data. Since the data showed normal distribution, ΔE values were analyzed using Two-Way ANOVA and Post-Hoc Tukey tests. All statistical analyses were performed using SPSS software version 17 (IBM, Armonk, NY, USA). A statistically significant level was determined as 5%.

## Results

**Fig. 2:**
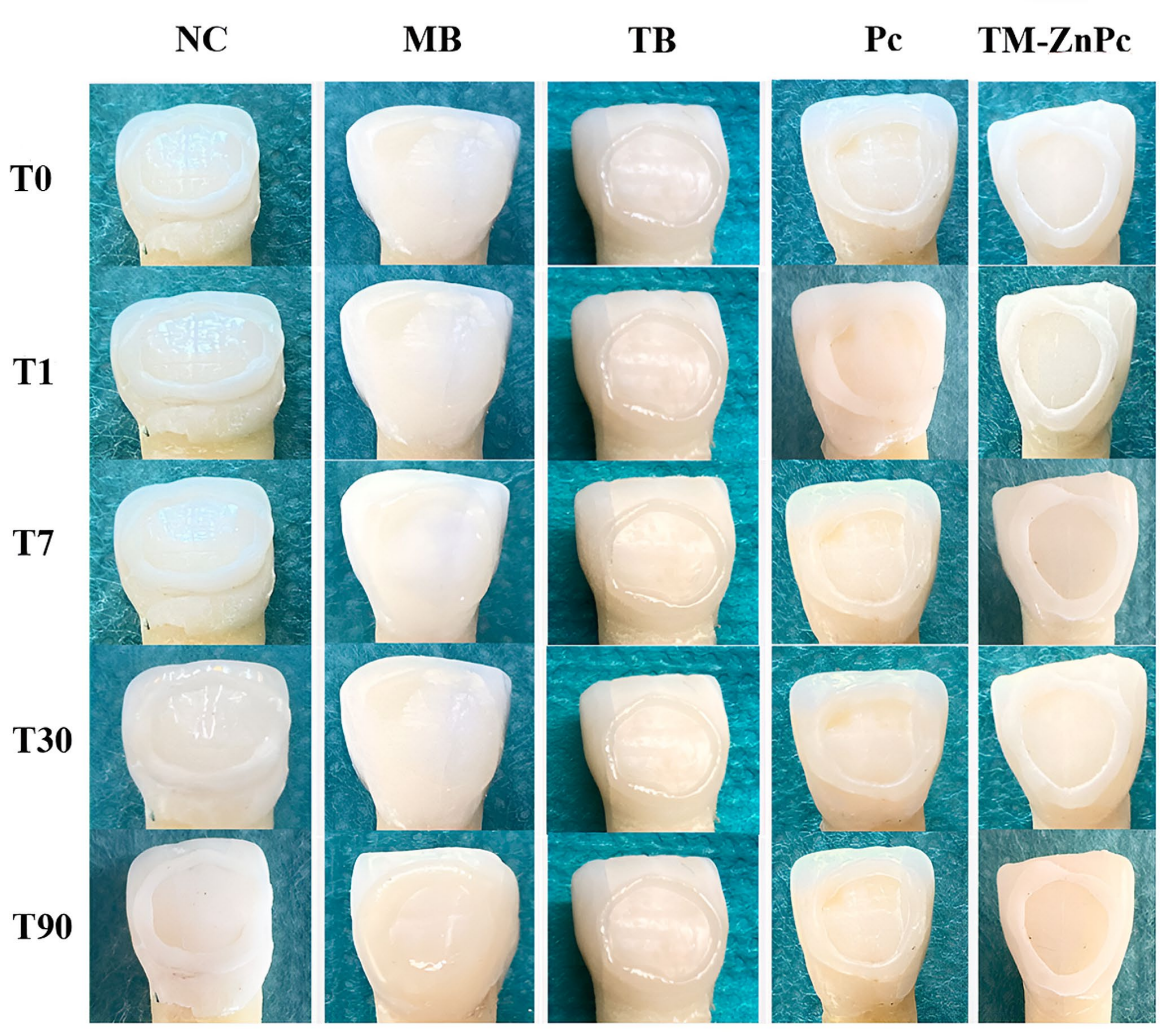
Photographs of a tooth belonging to each group taken at different time intervals

In the negative control group, no clinically detectable (ΔE ≤ 3.3) discoloration was observed at all time intervals evaluated (*p* > 0.05) Tooth discoloration was observed at 30 days after MB, TM-ZnPc applications and at 30 and 90 days after TB application compared to baseline (*p* < 0.05). Following the application of Pc, there were no significant differences observed in tooth discoloration across all evaluated time intervals (*p* > 0.05), as illustrated in Fig. 2 and summarized in Table 2.

**Table 2:** Evaluation of tooth discoloration among all FSs tested (mean ± standard deviation)

	C	MB	TB	Pc	TM-ZnPc
**T0-T1**	2,33 ± 0,63^ax^	14,92 ± 3,80^ay^	16,81 ± 4,38^ayz^	12,08 ± 4,86^ayq^	9,25 ± 5,04^abcq^
**T0-T7**	2,02 ± 0,60^ax^	15,25 ± 4,17^ay^	18,05 ± 2,53^ay^	12,02 ± 2,82^az^	9,77 ± 3,87^bz^
**T0-T30**	1,98 ± 0,72^ax^	12,45 ± 3,25^by^	14,88 ± 3,54^by^	12,31 ± 4,91^az^	6,94 ± 3,94^cx^
**T0-T90**	1,84 ± 0,68^ax^	15,45 ± 2,47^ay^	14,29 ± 4,85^by^	15,93 ± 8,02^ay^	11,60 ± 5,30^bdy^

* Significant differences between columns x, y, and z; significant differences between rows are indicated by a, b, and c

**Fig. 3:**
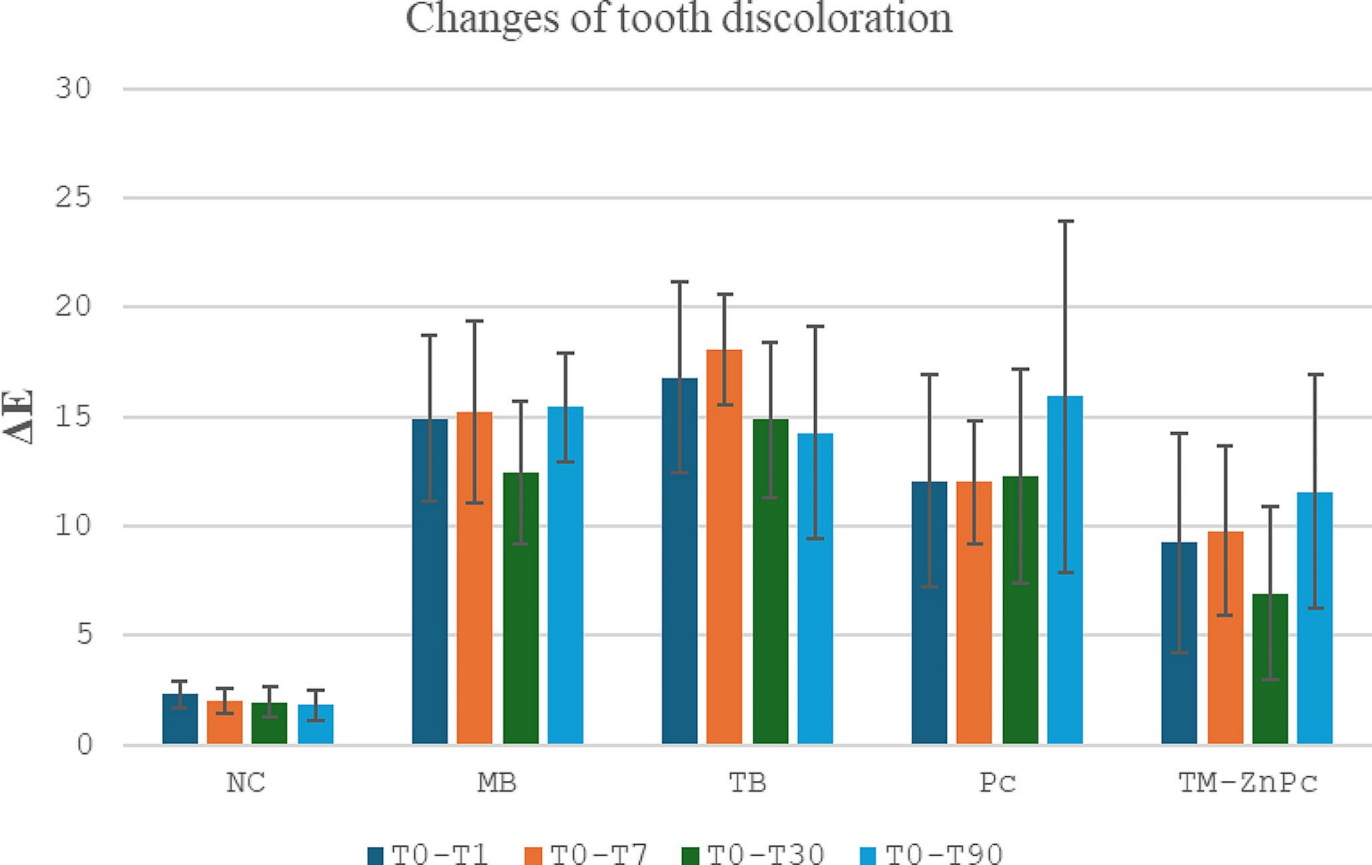
Changes of Tooth Discoloration

Tooth discoloration was determined to occur in all PSs tested compared to the negative control at all time intervals evaluated (*p* < 0.05) (Fig. 3). In the T0_T1 time interval, less tooth discoloration occurred in TM-ZnPc than in MB. There was less tooth discoloration after TM-ZnPc and Pc applications compared to TB (*p* < 0.05). In the time interval T0_T7, there was less tooth discoloration after TM-ZnPc and Pc applications compared to MB and TB (*p* < 0.05). In the time interval T0_T30, there was less tooth discoloration after TM-ZnPc application compared to MB TB and Pc (*p* < 0.05). There was no difference between the PSs tested in terms of tooth discoloration at time interval T0_T90 (*p* > 0.05) (Table 2).

## Discussion

There are many studies in the literature investigating the use of aPDT as an additional disinfection procedure due to its success in eliminating endodontic pathogens [19]. Therefore, the search for a PS without the potential for tooth discoloration in endodontics is currently ongoing [6]. In the literature, although there are studies investigating the potential of TB and MB for tooth discoloration [8, 32], it was determined that there were no studies investigating the tooth discoloration of Pc and TM-ZnPc, which show antimicrobial activity against endodontic pathogens. Therefore, the present study aimed to compare the tooth discoloration potential of TB, MB, TM-ZnPc, and Pc. The null hypothesis of the study was rejected because there was a difference between the PSs tested in the present study in terms of tooth discoloration.

Studies evaluating aPDT as an additional disinfection procedure in endodontic treatment have reported more antimicrobial efficacy of aPDT using MB, TB, TM-ZnPc, and Pc compared to a chemomechanical preparation alone [26, 33, 34]. However, having antimicrobial activity is not a sufficient justification for the clinical use of a PS. Presently, due to the increasing aesthetic concern, the effects on tooth discoloration are also an important criteria for clinical use. Therefore, in the present study, the effect of PSs on tooth discoloration investigated.

In the literature, it was determined that the pre-irradiation and irradiation time parameters of PSs are important factors in aPDT applications, but there is no consensus regarding the parameters. The pre-irradiation time of the PS varies between 1 and 5 min and the irradiation time varies between 30 s and 1 min [6, 19, 35–37]. Previous studies have determined the pre-irradiation time as 5 min and the irradiation time as 1 min, stating that this protocol provides sufficient microbial elimination. Therefore, the present study adopted these effective parameters to enhance clinical convenience [6, 38].

Different devices can be used to initiate the photochemical reaction in aPDT, including lasers, light-emitting diodes (LEDs), and gas discharge lamps (e.g. quartz-tungsten-halogen or xenon discharge lamps). Despite the advantages of lasers such as high potency and efficiency, they have the potential to cause damage to periapical tissues due to their high heat generation capacity and require high costs [25]. Filtered halogen lamps have the advantage that they can be spectrally filtered to suit any PS; however, they can damage periapical tissues due to their capacity to generate heat, in addition, the efficiency of halogen lamps can gradually decrease over time [39]. As LEDs move away from the light source, the light becomes more dispersed, reducing light power. To overcome this disadvantage, intracanal fibers are used. However, LEDs produce less heat than other light sources, offer a wide emission spectrum, are low cost, and easy to use. Considering these advantages, LED was used for photoactivation in the current study [25, 40–42].

In the literature, it has been reported that many methods such as visual comparisons, colorimetry, spectrophotometer, spectroradiometer, and digital image analysis are used in the evaluation of tooth color [43, 44]. Although visual color measurement using a color scale is a fast and cost-effective method, it is considered an inconsistent and subjective method due to factors such as lighting, age, gender, eye fatigue, and color vision deficiencies. Colorimeters are not considered as accurate as spectrophotometers and provide less information [45]. The advantage of digital image analysis is that the entire tooth surface can be evaluated, minimizing systematic error due to translucency and surface curvature [46]. However, under different lighting conditions, tooth colors that are different may appear the same due to the phenomenon of metamerism, which can be a disadvantage for these systems [44]. Since spectrophotometric devices perform contact-based measurements, the device must be held stationary during the measurement, which can be uncomfortable for the patient. Despite this disadvantage, spectrophotometric devices are considered the gold standard because they provide consistent results in repeated color measurements, their color-matching ability is more accurate than other methods, and they can measure following CIELab standards [47]. Considering these features, color measurements were performed using a spectrophotometric device.

As a result of the present study, no visible discoloration (ΔE ≤ 3.3) was observed in the teeth of the negative control. This is thought to be due to the favorable conditions in which the teeth were stored. As a result of this study, all PSs tested caused some degree of tooth discoloration. TB, MB, and TM-ZnPc caused tooth discoloration on Day 30 compared to baseline, but there was no difference in Pc in terms of undesirable discoloration over time. Consistent with the findings of our study, it was reported that TB and MB solutions applied with pre-irradiation times of 5 and 10 min caused tooth discoloration compared to baseline [8]. Similarly, a study, reported that after TB and MB solutions applied with a pre-irradiation time of 5 min, tooth discoloration occurred on day 60 compared to baseline [32]. The study indicated that after 5 min of pre-irradiation time on intraradicular dentin, tooth discoloration occurred due to MB [48]. Similarly, a study, reported that after TB and MB solutions applied with a pre-irradiation time of 5 min, tooth discoloration occurred on day 60 compared to baseline [32].

In a study [48], it was reported that after 5 min of pre-irradiation time on intraradicular dentin, MB resulted in tooth discoloration. In another study [49] was found that MB led to tooth discoloration in all groups (∆>3) following removal by various methods. The hydrophilic nature, low molecular weight, and cationic nature of MB and TB enhance binding to dental tissues [50]. Based on this information, MB and TB are thought to cause discoloration of dental tissues. There are no studies in the literature investigating the tooth discoloration potential of Pc and TM-ZnPc, so this part of our study results can not be directly compared with the results in the literature. As a result of our study, the result that TM-ZnPc caused discoloration on day 30 compared to the beginning, while Pc did not cause discoloration at all time intervals evaluated may be related to the presence of Zn in its content. There are many studies in the literature showing that zinc oxide eugenol-containing root canal sealants cause tooth discoloration [51, 52]. The discoloration potential of TM-ZnPc may be due to the zinc ion in its content.

In the present study, there was no difference between MB and TB regarding tooth discoloration at all time intervals evaluated. Similarly, a study reported that there was no difference between MB and TB in terms of tooth discoloration at 60 days by spectrophotometric analysis [29]. In contrast, another study reported that TB caused more tooth discoloration than MB after 5 and 10 min of pre-irradiation time [8]. Researchers [8] used Endo-PTC cream + 2.5% sodium hypochlorite with Tween 80 (detergent), urea peroxide, and carbomum to remove MB and TB [50]. The different methods used to remove residual PSs may have contributed to the different results of the study.

In the present study, TM-ZnPc and Pc caused less tooth discoloration compared to MB and TB on the 7th and 30th day. Since no studies are comparing MB and TB in the phenothiazine group with Pc and TM-ZnPc in the tetrapitol structure, the findings of our study in this direction were not compared with the data in the literature. The fact that Pc-derived PSs cause less tooth discoloration than PSs in the phenothiazine group may be due to their higher molecular weight and hydrophobic structure [53].

The fact that there was more tooth discoloration in the TM-ZnPc group than in the Pc group compared to the baseline may be due to the greater binding of the substituted Zn to collagen [54]. In the present study, the increased tooth discoloration in TB MB and TM-ZnPc compared to baseline may be due to their higher binding to organic tissues [55–57]. Compared to baseline, tooth discoloration was observed on the 30th day after MB and TM-ZnPc application and on the 30th and 90th day after TB application. No significant difference was observed in terms of tooth discoloration at all periods evaluated after Pc application. Therefore, we consider that Pc is a more safe PS in terms of tooth discoloration potential.

It has been reported that irrigation solutions and root canal sealers used in endodontics cause discoloration [58]. The present study was designed solely to investigate the coloration caused by the PS tested. However, the interaction of residual photosensitizers with EDTA, NaOCl or root canal sealer to be used after the irrigation in endodontic treatment may also cause discoloration. Therefore, there is a need for study designs that will investigate discoloration in obturated teeth after aPDT is performed with PSs [59, 60].

## Conclusion

Under the limitations of this study, all tested PSs were found to cause some degree of tooth discoloration. Further studies are needed to evaluate the efficacy of the tested PSs under clinical conditions with longer follow-up periods. This is the first study to investigate the tooth discoloration potential of TM-ZnPc and Pc. Therefore, further studies are needed to evaluate the tooth discoloration potential of TM-ZnPc and Pc from different aspects.
